# Biodiversity of Skin Microbiota as an Important Biomarker for Wound Healing

**DOI:** 10.3390/biology12091187

**Published:** 2023-08-30

**Authors:** Caglar Ersanli, Athina Tzora, Chrysoula (Chrysa) Voidarou, Stylianos Skoufos, Dimitrios I. Zeugolis, Ioannis Skoufos

**Affiliations:** 1 Laboratory of Animal Science, Nutrition and Biotechnology, Department of Agriculture, University of Ioannina, 47100 Arta, Greece; c.ersanli@uoi.gr (C.E.); jskoufos@uoi.gr (I.S.); 2 Laboratory of Animal Health, Food Hygiene and Quality, Department of Agriculture, University of Ioannina, 47100 Arta, Greece; xvoidarou@uoi.gr (C.V.);; 3 Regenerative, Modular & Developmental Engineering Laboratory (REMODEL), Charles Institute of Dermatology, Conway Institute of Biomolecular and Biomedical Research, School of Mechanical and Materials Engineering, University College Dublin, D04 V1W8 Dublin, Ireland; dimitrios.zevgolis@ucd.ie

**Keywords:** wound healing, skin microbiota, manipulation strategies, coagulase-negative *S. aureus*, *S. aureus*, *P. aeruginosa*, *Lactobacilli*

## Abstract

**Simple Summary:**

Wounded skin can naturally be repaired by a mechanism called wound healing. Human skin is a habitat of various pathogenic and commensal bacteria. While these bacteria are in balance in healthy skin, they can lose the balance by wounding, which leads to delay in the wound-healing process. Moreover, commensal and pathogenic bacteria inhabit skin tissue and have constant communication with the immune system, which can increase and decrease the healing efficiency, respectively. This indicates that cutaneous bacteria have important effects on wound healing. Herein, we discuss some important bacteria (coagulase-negative *Staphylococci* (CoNS), *S. aureus*, *P. aeruginosa*, and *Lactobacilli*) present in human skin, the effects of communication of bacteria with the immune system and epithelial cells on wound healing, and the identification techniques and manipulation strategies of the bacterial population in wounded skin tissue.

**Abstract:**

Cutaneous wound healing is a natural and complex repair process that is implicated within four stages. However, microorganisms (e.g., bacteria) can easily penetrate through the skin tissue from the wound bed, which may lead to disbalance in the skin microbiota. Although commensal and pathogenic bacteria are in equilibrium in normal skin, their imbalance in the wound area can cause the delay or impairment of cutaneous wounds. Moreover, skin microbiota is in constant crosstalk with the immune system and epithelial cells, which has significance for the healing of a wound. Therefore, understanding the major bacteria species in the cutaneous wound as well as their communication with the immune system has gained prominence in a way that allows for the emergence of a new perspective for wound healing. In this review, the major bacteria isolated from skin wounds, the role of the crosstalk between the cutaneous microbiome and immune system to heal wounds, the identification techniques of these bacteria populations, and the applied therapies to manipulate the skin microbiota are investigated.

## 1. Introduction

Cutaneous wound healing is a complicated and well-organized natural repair process that comprises four stages: hemostasis, inflammation, proliferation, and remodeling [1]. Skin damage, inducing a wound, allows organisms from foreign bodies to penetrate through the wound site [2,3]. In other words, a wound procures an occasion for both commensal and pathogenic microorganisms to access underlying tissue, then grow and colonize after reaching ideal conditions [4], which may cause further impairment in wound healing.

The complex and rich ecosystem of skin microbiota (microbiome) arises from diverse microorganisms, i.e., bacteria, fungi, viruses, and yeasts [5], and has a significant role in the protection of skin tissue and ensuring hemostasis [6,7]. The bacteria inhabiting the cutaneous microbiota can be classified as commensal and pathogenic. Pathogenic bacteria are a harmful bacteria type that can directly be transmitted to the host tissue and lead to infection. In contrast, commensal bacteria can supply essential nutrients to the host tissue and benefit in fighting infection. Although bacteria are the most abundant microorganisms in skin microbiota, only about 25% of them can move through the deeper skin layers [8], being important players in skin physiology and disease processes [9]. Commensals present in skin microbiota have been determined as beneficial with their ability to originate immune response thanks to their communication with cutaneous cells such as keratinocytes and fibroblasts [10,11]. These types of bacteria have advantageous effects on wound healing by providing a barrier function for the skin and combating pathogenic microorganisms [12]. In contrast, pathogenic bacteria may give rise to delayed or impaired wound healing [13,14] by leading to infection in the wound site.

The crosstalk between the cutaneous microbiome, immune system, and epithelial cells is evaluated significantly for tissue repair and regeneration in vertebrates [15]. The harmony among all these provides an efficacious approach to wound healing and an invasive system by possible pathogens in equilibrium [16]. However, in the case of the disequilibrium between commensals and pathogens, cutaneous diseases may appear. In this perspective, understanding of the communication between the microbiome and the immune system as well as the identification and manipulation of the microbiome by several approaches has become prominent as an alternative solution for cutaneous wound treatment. In this review, we first discuss the abundant pathogenic and commensal bacteria that inhabit a wound. Moreover, we examine the effects of the crosstalk between skin microbiota and the immune system, as well as the identification and manipulation of skin microbiota, to come up with a different perspective for cutaneous wound healing mechanisms.

## 2. The Abundant Bacteria Implicated in Wound

Both aerobic and anaerobic microorganisms that constitute skin microbiota inhabit the skin surface soon after birth in a dynamic correlation with the host [17]. Although the bacteria protect the skin balance, they may penetrate through the underlying skin tissues when their continuity is broken by intrinsic and/or extrinsic factors. Thereafter, these penetrated bacteria can lead to the formation of colonization or contamination. Contamination is the presence of potentially pathogenic microorganisms in the wound area, whilst colonization is the existence of replicating microorganisms with no damage to the wound. However, critical colonization is the threshold that may delay the healing of the wound due to the high number of bacterial counts. Local infection with critical colonization, and proliferation of microorganisms, as well as local tissue reactions, can cause generalized host reactions, thereby an invasive infection [18]. Therefore, understanding the species and effects of the bacteria in skin tissue is important to develop solutions for infected wounds.

Even though many studies have revealed the positive effect of the skin microbiota in wound healing either by modulating immune response or preventing pathogen invasion, the precise relationship between commensal microbiota and impaired wound healing remains unclear [19]. Some studies state that regardless of the destination between friend and foe, the skin microbiota tends to play a negative role in wound healing in different ways such as the elevation of pro-inflammatory mediators [20]. For instance, the persistence of bacteria in wounds impairs the healing process by elevation of pro-inflammatory cytokines such as interleukin-1 and tumor necrosis factor-alpha that in turn cause increased levels of matrix metalloproteinases (MMPs), a decreased level of tissue inhibitors to the MMPs, and decreased production of growth factors [21]. Cytokines are important signaling proteins that modulate fundamental pathophysiological and hemostatic processes, e.g., wound healing, by inducing downstream signal transduction pathways via specific cytokine receptors [22,23]. Therefore, cytokines can regulate the function of other receptors such as sodium channels, as well as the transient vanilloid receptors, and modulate the hemostatic process. Hence, a significant reduction in the number of pathogenic microbes using an appropriate antimicrobial agent is vital in regularizing wound healing. Moreover, satisfactory wound repair is possible only when the infection is brought under control. In other words, acceleration in the wound healing process is proportional to the reduction of the number of pathogenic microbes in the wound bed [24,25,26,27].

**Figure 1:**
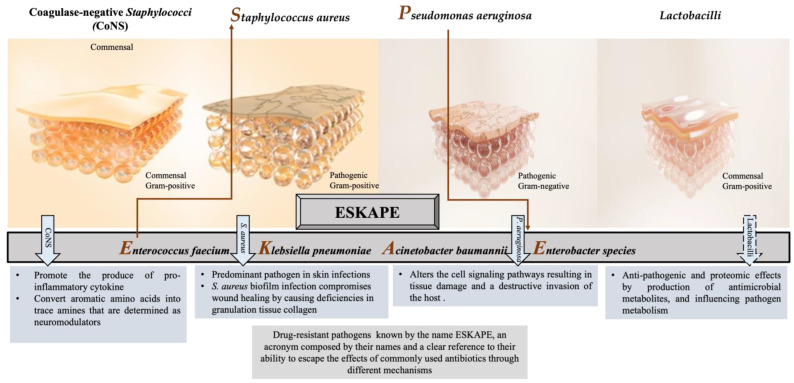
Different responses of the most often isolated bacteria to the process of wound healing.

Numerous studies have reported that the most common pathogens associated with wound infections are *Staphylococcus aureus* (*S. aureus*); *Pseudomonas aeruginosa* (*P. aeruginosa*); *Escherichia coli* (*E. coli*); coagulase-negative Staphylococci (CoNS), i.e., *Staphylococcus epidermidis* (*S. epidermidis*); *Streptococcus pyogenes* (*S. pyogenes*); *Klebsiella* spp.; and *Proteus* spp. [28,29,30,31,32]. In chronic wounds, *S. aureus*, followed by *P. aeruginosa*, is the most common isolated microorganism [2,33,34], inhibiting wound healing, and is considered dominant [35,36]. However, many other commensal species have been isolated from cutaneous wounds, such as lactobacilli, which might have a positive therapeutic effect. Herein, we focus on the effect of CoNS, *S. aureus*, *P. aeruginosa*, and Lactobacilli on the wound-healing process, as illustrated in Figure 1.

### 2.1. Coagulase-Negative Staphylococci (CoNS)

*Staphylococci* (mainly *S. epidermidis*, *S. haemolyticus*, *S. hominis*) are generally abundant bacteria that inhabit normal skin flora. Some CoNS species demonstrate a promoting influence on the wound as well as preventing a chronic process. For example, commensals of the human skin microbiota, such as *S. epidermidis*, stimulate IL-17+ CD8+ T cells, which are able to produce the pro-inflammatory cytokine IL-17A and restrict pathogen intrusion [37]. Moreover, *S. caprae* inhibits the quorum sensing (QS) of *S. aureus* by forming an autoinducing peptide, which leads to the expression of diverse virulence genes [38]. On the other hand, the lantibiotics gallidermin and epidermin, which are produced from certain CoNSs or antimicrobial peptides, displayed the blockage of cell wall biosynthesis in Gram-positive bacteria that might cause the inhibition of *S. aureus* [39].

*S. epidermidis* has been getting attention with its triggering mechanisms that reduce harmful microbes and encourage wound healing in the acute phase among the other CoNSs [40]. Recent studies have shown that *S. epidermidis* plays an active role in skin immunity, protecting from the invasion of Gram-positive and Gram-negative pathogens [41]. The SadA-expressing *S. epidermidis* strains that commonly inhabit human skin and gut microbiota [31,36] can convert aromatic amino acids into trace amines that are determined as neuromodulators by interacting with diverse adrenergic receptors. The wound-healing process is enhanced thanks to this biological reaction that induces the increase in keratinocyte migration, re-epithelialization rate, and extracellular-signal-regulated kinase level [42,43]. On the other hand, trace-amine-producing skin commensals can contribute to the acceleration of wound healing by suppressing the adrenaline and represent a promising therapeutic option [44].

### 2.2. Staphylococcus aureus

*S. aureus* is one of the most common and predominant pathogens with approximately 65% prevalence, involved in skin infections worldwide, which can cause persistent infections in chronic wounds with adverse effects [45]. This bacterium was first described by the Scottish surgeon Alexander Ogston as an isolate from a wound, and he demonstrated its significance as a pus pathogen [46]. As a commensal or opportunistic pathogen, *S. aureus* has been isolated from various reservoirs with a tendency to disseminate among them, such as humans, animals, and the environment [47,48,49], revealing genetic relatedness and sometimes threatening public health [50,51].

*S. aureus* is a pathobiont for humans and animals, leading to the emergence of more virulence and multidrug-resistant strains. Hence, this situation makes *S. aureus*-caused wound infections dangerous and requires the development of new prevention models and treatment strategies [52]. Because of the invasion, this bacterium can cause a variety of diseases, ranging from minor skin and soft tissue infections such as impetigo, folliculitis, and abscesses, to life-threatening systemic infections such as sepsis, endocarditis, or toxic shock syndrome [53]. It is also worth mentioning that the biofilm formation ability of most *S. aureus* strains is one of the specific virulence factors enabling them to adapt to the chronic wound environment [54].

Some *S. aureus*-strain-infected wounds may be challenging or almost impossible to treat due to their mostly antibiotic-resistant and high virulence nature, contributing to the pathogenicity of the host [55]. This high virulence allows it to escape the immune system’s reaction and thus the antibiotic activity. For instance, the existence of a β-lactamase enzyme in *S. aureus* enables it to break the ring of β-lactam antibiotics, making the antibiotics inactive [56]. In particular, some strains are resistant to methicillin as well as to other antibiotics. Methicillin resistance occurs due to the acquisition of mecA or mecC genes by previously susceptible strains that are consequently called methicillin-resistant *S. aureus* (MRSA) [57], followed by resistance to all β-lactam antibiotics, except for the fifth-generation cephalosporins [32,58]. MRSA is responsible for nosocomial infections, which are more difficult to treat. Patients with infections caused by MRSA have a much greater mortality risk than methicillin-sensitive strains [59].

As a consequence, the emergence and the unceasing spread of multi-resistant *S. aureus* strains, such as methicillin or vancomycin-resistant *S. aureus*, complicate the treatment of *Staphylococcal* infections with detrimental impact on global health and the economy. Therefore, the WHO has justifiably enlisted *S. aureus* as one of the major health threats in the so-called “post-antibiotic era” [60]. Thus, the development of new antibiotics and alternative prophylactic or therapeutic strategies have become inevitable to combat *S. aureus*, as well as other five ESKAPE bacteria (Figure 1), which possess multidrug resistance and high virulence [61,62].

### 2.3. Pseudomonas aeruginosa

*P. aeruginosa* is a Gram-negative bacterium included in the ESKAPE bacteria with concerns for public health, like *S. aureus*, and has a place in the concept of the “one health approach” [63]. It elicits prolonged hospitalization with increased morbidity and mortality rates [64] as a common opportunistic pathogen that causes several chronic, treatment-resistant infections in humans, i.e., certain skin, respiratory, and urinary tract infections [65,66]. Moreover, it has been isolated from patients with burn wounds, cystic fibrosis, acute leukemia, organ transplants, and intravenous drug addiction [67]. This bacterium penetrates wounds, holding the ability to form intact biofilms and subsequently degrading the extracellular matrix and altering the cell signaling pathways, resulting in tissue damage and a destructive invasion of the host [68,69]. *P. aeruginosa* has been reported as a detrimental pathogen during the past two decades, with grounds for 10 to 20% of infections in most hospitals, being determined as one of the twelve prior pathogens that pose the greatest threat to human health according to the WHO [62]. In 2017, it was estimated that 32,600 infections were caused by multidrug-resistant *P. aeruginosa* among hospitalized patients, and 2700 deaths occurred in the US [70].

The emergence of multidrug-resistant *P. aeruginosa* resulting in the persistence and non-response to clinical treatment of infectious diseases such as infected wounds has been referred to by several studies [71,72,73]. *P. aeruginosa* has been evaluated as resistant to diverse antibiotics, such as β-lactams, aminoglycosides, quinolones, and sulfonamides [74,75]. This resistance is derived from its excellent ability to select chromosomal mutations and acquire resistant genes, bearing multiple antimicrobial resistance mechanisms, which led *P. aeruginosa* to become one of the most difficult bacteria to treat [76,77]. 

Different mechanisms are involved in the expression of resistance of *P. aeruginosa* coming from innate and acquired ways. Innate resistance is related to an overexpressed efflux pump and low permeability of the outer membrane of the bacterium [78]. Acquired resistance involves the acquisition of a resistance gene or mutation in genes encoding porins, efflux pumps, penicillin-binding proteins, and chromosomal β-lactamase, all contributing to resistance to β-lactams, carbapenems, aminoglycosides, and fluoroquinolones [79]. *P. aeruginosa* strains have also been found resistant to aminoglycosides carrying the mexXY genes that induce the modification of aminoglycoside enzymes [80]. On the other hand, the genes crpP and qnrVC1 have been identified in clinical isolates of *P. aeruginosa*, which demonstrated resistance to fluoroquinolone [81]. Hence, all these concerns become *P. aeruginosa*-infected wounds, making developing treatment strategies vital.

### 2.4. Lactobacilli

In general, Lactobacilli are defined as beneficial bacteria and constitute a valuable member of an organs’ microbiota where they are located [82]. They are endogenous inhabitants of healthy skin, contrary to inflammatory skin, which is often associated with disturbed skin microbiota. The abundance and the diversity of Lactobacilli in the skin depend on the phylum of the host as well as on the anatomical areas [83].

The potential benefit of Lactobacilli as a skin habitat is based on the competition that exerts against skin pathogens through adhesion inhibition, production of antimicrobial metabolites, and influencing pathogen metabolism. Furthermore, their metabolites have proven to be immunomodulators, reducing excessive skin inflammation. In accordance with these effects, the functions of Lactobacilli as a skin barrier have already been tested in several clinical trials, making them a primarily promising alternative agent for promoting skin health [84]. Beyond the typical skin commensals, other bacterial species such as Lactobacilli might be beneficial for wound healing, either by their lysate that increases the migration and proliferation of keratinocytes or by the production of organic acids that act against pathogens and inhibit biofilm formation on wounds [85,86,87]. Several studies have shown that the application of probiotics causes a reduction in wound infections [86,88], especially when used as an adjuvant to antibiotic therapy. *Lactobacillus plantarum*, *Lactobacillus casei*, *Lactobacillus acidophilus*, and *Lactobacillus rhamnosus* are the most commonly used probiotics for most studies, which are well-known strains of the species with their positive effects [85]. All in vitro studies with these probiotics revealed successful inhibition of chosen skin or wound pathogens. Moreover, within the scope of in vivo studies on mice, rats, and rabbits, probiotics exhibited strong opportunities for counteracting wound infections caused mainly by *S. aureus* and *P. aeruginosa*. In regards, clinical studies generally showed a slight or statistically significant lower incidence of infections for patients using probiotics [85]. In particular, antimicrobial, quorum sensing, anti-biofilm, and adhesion assays were carried out with several probiotic strains and tested against selected skin pathogens. For instance, the tested Lactobacilli except for *Lactobacillus delbrueckii* exerted antimicrobial activity against skin pathogens, mainly due to organic acid production. On the other hand, most of them could prevent biofilm formation by selected pathogens [87].

Although probiotic Lactobacilli have a ‘generally regarded as safe’ (GRAS) status for food, the potential risks of live probiotics entering the bloodstream through breached skin has not been assessed. Hence, as opposed to previously mentioned studies, some investigations were conducted with the lysates of these probiotic strains as they could represent a safer alternative to the use of live bacteria in a wound situation. Moreover, the use of lysates may be of more utility to potential wound care manufacturers than live bacteria by overcoming the logistical requirements of maintaining viable bacteria within a formulation or wound dressing [89].

## 3. Communication between Skin Microbiota, Immune System, and Epithelial Cells

In vertebrates, regularly, all anatomical surfaces that communicate with the environment are colonized by microbes that compose the microbiome. Therefore, the study of the physiological and metabolic effects of the human microbiome on multicellular organisms regarding both health and disease concerns has become prominent. 

The largest organ of the human body, the skin, is home to approximately 10^12^ bacterial cells [90,91] that comprise the skin microbiota, thanks to its immediate interface with the environment [17]. The skin microbiome, as mentioned, comprises a diverse population of fungi, bacteria, archaea, viruses, and sometimes parasites in close interaction with vertebrate hosts [92], and it contributes to the barrier function and hemostasis of skin tissue in various ways. For instance, the secretion of protease and lipase enzymes are involved in the desquamation and lipid surface degradation processes, respectively. Likewise, free fatty acid and sebum formation take place in the pH regulation of the skin tissue [93]. 

The skin microbiota has significant roles, such as the production of biofilms, bacteriocins, and quorum sensing [94,95]. It protects the skin tissue against pathogenic microorganisms by competition [96,97] and leads the production of antimicrobial peptides by virtue of commensal bacteria [39,98], which are in crosstalk with the immune system continuously that may be beneficial for the healing of the wound [99].

**Figure 2:**
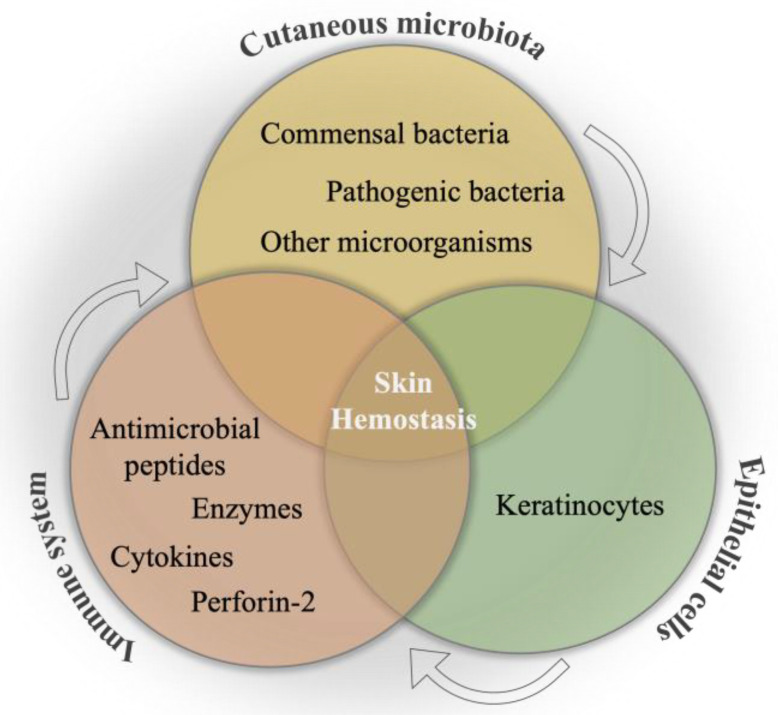
Schematical illustration of the relation between cutaneous microbiota, immune system, and epithelial cells for skin hemostasis.

The internal communication between skin microbiota, epithelial cells, and the immune system is a primary mechanism to combat pathogenic invasion, as well as to ensure the maintenance of the skin commensals [14], which is schematically illustrated in Figure 2. This primary interaction has been initiated by keratinocytes toward the binding of pathogen-associated molecular patterns to pattern recognition receptors, resulting in the release of antimicrobial peptides as an inhibitory agent to diverse pathogens in the infection area. Moreover, the efficacy of various impacts of different microorganisms on the immune system has been determined as an important phenomenon in wound healing. For instance, the colony formation in wound bed by *S. epidermidis* has led to an increase in the expression of the cytokine, interleukin 1α (IL-1α). This cytokine is responsible for contributing to skin inflammation and host defense, which directly promotes wound healing [100]. In a study, the communication among commensal organisms, pathogens, and keratinocytes was investigated using polymicrobial biofilms formed by a mixture of commensal strains (*S. epidermidis* and *M. luteus*) and pathogens (*S. aureus* and *P. aeruginosa*). The commensals demonstrated the reduction of the damage caused by pathogens on the keratinocyte monolayer via degrading biofilm thickness and forming a layer between the keratinocytes and pathogens [101].

The innate immune protein Perforin-2, which is expressed by keratinocytes, is a key element for the inhibition of several Gram-positive and Gram-negative bacteria in the wound area. Even though it has proven inhibition activity on bacterial cells, in a study, *S. aureus* caused infection by inhibiting the expression of the Perforin-2 protein, which caused the delay of wound closure [102]. On the other hand, in vitro MRSA and *P. aeruginosa* caused polymicrobial wounds, demonstrating a delay in the reepithelization of the wound due to the downregulation of keratinocyte growth factor-1 expression, which is vital to promoting the proliferation and migration of keratinocytes [103]. In contrast to those negative impacts, skin commensals such as *S. epidermidis* were evaluated with their promoting effect on wound recovery, owing to their ability to induce diverse antimicrobials [11]. Alongside the inhibitory action of *S. aureus* on Perforin-2 expression, skin commensals are able to inhibit bacterial wound infections by altering the wound environment as well as skin tissue.

The microbiome composition of a wound may vary according to the wound type and influence its healing potential directly. Several studies have proved that most nonhealing wounds have a polymicrobial nature [30]. Both intracellular and extracellular bacteria exist in a biofilm, indicating several effects on the wound. Therefore, interactions between skin commensals and pathogens have gained importance in understanding wound healing regulation. As previously mentioned, *S. aureus* is one of the most abundant bacteria on wounds that mostly shows antimicrobial resistance, hence leading to delayed or impaired wound healing. Some species of CoNS are able to inhibit the *S. aureus* colonization or *S. aureus*-related biofilm formation. S. lugdunensis is able to prevent *S. aureus* colonization by producing the antibiotic lugdunin [104]. Likewise, *S. hominis* demonstrates the synergetic effects with antimicrobial peptide LL-37, owing to the lantibiotics they synthesize, hence prohibiting the colony formation of *S. aureus* [39]. It was also shown that serine protease glutamyl endopeptidase expressing *S. epidermidis* killed the *S. aureus* cells by promoting keratinocytes for the production of antimicrobial peptides [105]. Although some species of skin bacteria commensals seem beneficial and prominent for fighting *S. aureus*-caused infections, coproporphyrin III produced by *Propionibacterium acnes* led to the biofilm formation of *S. aureus* [17].

## 4. Identification of the Skin Microbiota

The identification of patients’ ‘core’ microbiome can help to understand its role in the pathogenesis of disease and subsequently discover new therapeutic strategies through microbial intervention [106]. The culture-dependent, denatured, and temperature gradient gel electrophoresis (DGGE and TGGE), metagenomic, and culturomic are techniques commonly used for identifying the composition of the skin microbiome (Table 1).

The bacterial diversity of wounds has traditionally been detected by culture-dependent methods immediately after the collection of samples from the wound area. The samples are exposed to specific growth media to let the unknown bacteria grow. This cost-friendly and easy-to-study technique depends on the growth conditions and media according to the specific microorganisms [107]. Even though culture methods tend to select microorganisms that can grow under certain laboratory conditions, the detection of non-culturable microorganisms becomes limited.

The advanced sequencing methodologies, also called metagenomic techniques, provide more clarity as well as unbiased profiling on wounded skin microbiomes compared to culture techniques with higher taxonomical determination and functional investigations of bacteria, e.g., antibiotic resistance. On the other hand, these techniques are beneficial for the detection of bacterial cells in the formed biofilms on the wound area, which are highly difficult to reveal by culturing techniques [110,111]. These amplicon-based methods demonstrate more accuracy for bacterial diversity in collected samples than bacteria that grow in an artificial environment. Hence, culture-independent techniques have revealed many recent strains that previously have not been detected via culturing methods. In other words, wound microbiomes have started to show more diversity than previously suggested. For example, in a study, researchers compared culture and advanced sequencing diagnostic methods on bacterial diversity in wound microbiota by sampling 168 different wounds. The molecular diagnosis technique identified up to 33 different bacteria in the individual wound, whilst up to 3 bacteria were identified by culture-dependent methods [112].

Next-generation sequencing-based techniques can characterize the wound bioburden, which provides an important understanding of the role of the microbiota in regulating the wound healing process, as well as clinical outcomes including infection-caused complications [18]. These amplicon-based sequencing techniques with the advantage of 16s rRNA, which exists in all prokaryotes, contrary to eukaryotes, and is conserved among different bacterial species, greatly expand the knowledge of wound microbiota. Moreover, analyses focusing on fungal ITS gene sequencing provide additional insights into the impacts of non-bacterial microorganisms in chronic wounds.

Whole-genome sequencing (WGS) is a versatile analysis to determine a microbiome that represents not only the wound bioburden but also discriminates the microbial diversity of healthy and wounded skin with the beneficial effects to prevent and/or treat the wound. In comparison with published sequencing analyses of normal human skin microbiota, chronic wounds have more anaerobes, Gram-positive cocci, and Gram-negative rods, and fewer commensals, e.g., *Propionibacterium* [113]. In particular, *Staphylococcus*, *Pseudomonas*, and *Corynebacterium* are mostly isolated species from chronic wounds. The WGS, also called shotgun metagenomics sequencing, also contributes to the understanding of clinically relevant insights into the effects of strain level diversity, mechanisms of virulence, and responses to therapeutics as well as specific essential genes [114,115].

## 5. Manipulation of the Skin Microbiota

**Figure 3:**
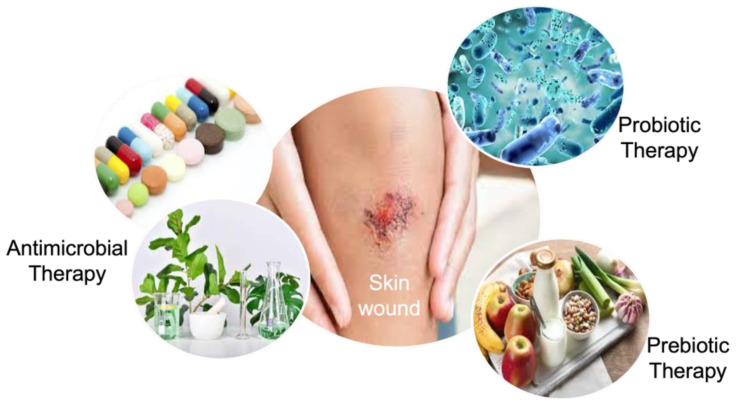
The manipulation strategies of the wounded skin microbiome.

Most skin conditions such as wound infections are related to the instability in skin microbiota [114]. Microorganisms in wounded skin microbiota may delay or block the natural healing process as well as inflammation, even if the bacterial infection has not initiated wounding. Therefore, the dynamic skin microbiota presents an adequate platform for wound-healing treatment [116,117]. The manipulation of the skin microbiota has been studied by antimicrobial and prebiotic/probiotic therapies to accelerate the healing of the wound (Figure 3).

Antimicrobial therapy is the most studied manipulation method for wound healing by aiming for the inhibition and/or reduction of a considerable number of bacteria in the wounded skin microbiota. Antibiotics are a well-known and extensively used antimicrobial agent for this purpose. For instance, in a retrospective study that identified wounds frequently co-infected by *S. aureus* and *P. aeruginosa*, ampicillin indicated the higher resistance on 134 isolates, corresponding to 56.1%, followed by gentamicin, penicillin, trimethoprim/sulfamethoxazole, and piperacillin, in that order [118]. However, the misuse and overuse of antibiotics have led to the generation of drug-resistant pathogens [119,120], and antimicrobial resistance concerns were reported as one of the top global public health threats facing humanity by the World Health Organization [121]. Therefore, exploring alternative and cost-effective antimicrobials based on traditional plant-based medicines has become prominent [122]. Plant-based antimicrobials (e.g., essential oils, herbal extracts) have been determined as effective alternatives to accelerate the natural wound healing process, as well as tissue regeneration in the wound area due to the broad range of natural bioactive compounds contained in herbs [123,124,125]. Moreover, approximately 73% of pharmaceutical products include ingredients derived from natural products [126]. Recent studies have shown that medicinal plants improve wound healing in diabetic, infected, and opened wounds. For example, *Moringa oleifera* seeds loaded with n-hexane hydrogels inhibited the growth of *S. aureus*, *E. coli*, and *P. aeruginosa*, showing almost complete closure of excision wounds in a male Swiss albino mice model [127]. In another study, composite polymeric hydrogels, including *Didymocarpus pedicellatus* plant extract, gave rise to complete wound closure in diabetic rats [128]. However, plant-based antimicrobials are generally incorporated into a biomaterial in order to improve their stability, sensitivity, and on-target inability.

Probiotic and prebiotic therapies are another manipulation method for wounded skin microbiota. This manipulation technique aims to increase the number of beneficial microorganisms in the wound area by using living organisms (probiotics) [129], specific nutrients (prebiotics), or a special concept that favors the idea of the utilization of collage-type material infused with probiotics/prebiotics as a means of prevention and/or treatment options in skin disease [130,131]. Probiotics have demonstrated a reduction in bacterial load and promote wound healing [132]. Contrary to antibiotics, probiotic and prebiotic therapies are generally studied in vivo. In a clinical study with 80 patients who had second- or third-degree burn wounds, topical administration of *Lactobacillus plantarum* significantly accelerated the healing rate to 17% higher than the standard silver sulfadiazine for 3–7 days post-burn [133]. In comparison with probiotics, the prebiotic-based approach is an indirect method with the advantage of the reduced chance of immune response of skin tissue. However, they may also affect the low abundance of non-targeted bacteria in the skin microbiome [134].

## 6. Conclusions

Bacteria inhabit cutaneous microbiota, which is in balance under normal conditions, and have an important role in the natural wound healing process. The skin microbiota can easily become imbalanced after wounding of the skin tissue and enhance or diminish the healing process due to several reasons such as microbial load, dominant bacteria species, and communication between the immune system. Therefore, the understanding of the major bacteria present in the wound bed, their communication between the immune system and epithelial cells, their identification process, and their manipulation strategies to re-balance the cutaneous microbiota have come to the forefront. We believe that this review study, investigating various perspectives about the role of the skin microbiota under wounding conditions, can provide a way to develop new strategies for wound healing.

## Figures and Tables

**Table 1:** The identification techniques for skin microbiota.

Technique	Definition	Benefits	Limitations	References
Culture	Isolation and growth of microorganisms on selective media	Cost effective Semiquantitative approach Easy to analyze data	No detection of new microorganisms Difficult to detect embedded bacteria in biofilm Less sensitive for microorganism detection than DNA-based approaches Dependent to microorganisms growing in an artificial environment	[107]
Temperature and denatured gradient gel electrophoresis (TGGE and DGGE)	Gel separation of 16s rRNA amplicons via temperature or denaturant	Ability to detect broad microflora Easy to analyze data Cost effective	Limited to detection of new microorganisms Time-consuming approach Gel may melt during the process that becomes genetic material in unexpected forms	[107]
Metagenomics	Parallel sequencing of numerous different bacteria by partial 16s rRNA or whole genome	Do not require growth and isolation of bacteria Ability to analyze massive samples Generic database for references Ability to detect uncultured microorganisms Bacteria embedded in a biofilm can be detected Low operational costs	PCR-amplification-dependent results Possibility of contamination of human DNA during the experiment High operational costs Cannot individuate live and dead microorganisms	[107,108,109]
Culturomics	Sample culturing followed by numerous parallel sequencings of partial 16S/18S rRNA amplicons	Take advantage of both culture and metagenomics approaches Can individuate live and dead microorganisms Detection of new microorganisms includes viruses Quantitative approach Phylogenetic assessment	Complex data analyses Not user friendly High operational costs	[107,108,109]

## Data Availability

Data are contained within the article.
